# Complex Variation in Afrotropical Mammal Communities With Human Impact

**DOI:** 10.1002/ece3.71331

**Published:** 2025-05-26

**Authors:** Deogratias Tuyisingize, Lars Kulik, Delagnon Assou, Diorne Zausa, Solange Kamga, Onella Mundi, Stefanie Heinicke, Inza Kone, Samedi Jean Pierre Mucyo, Tenekwetche Sop, Christophe Boesch, Colleen Stephens, Anthony Agbor, Samuel Angedakin, Emma Bailey, Mattia Bessone, Charlotte Coupland, Josephine Head, Tobias Deschner, Paula Dieguez, Villard Ebot Egbe, Anne‐Céline Granjon, Thurston Cleveland Hicks, Sorrel Jones, Ammie K. Kalan, Kevin E. Langergraber, Juan Lapuente, Kevin C. Lee, Laura K. Lynn, Nuria Maldonado, Maureen S. McCarthy, Amelia Meier, Lucy Jayne Ormsby, Alex K. Piel, Lilah Sciaky, Volker Sommer, Fiona A. Stewart, Erin G. Wessling, Jane Widness, Roman M. Wittig, Pauline Strohbach, Mimi Arandjelovic, Yntze van der Hoek, Hjalmar S. Kühl

**Affiliations:** ^1^ Dian Fossey Gorilla Fund Musanze Rwanda; ^2^ Senckenberg Museum for Natural History Görlitz, Senckenberg—Member of the Leibniz Association Görlitz Germany; ^3^ Laboratory of Ecology and Ecotoxicology (LaEE) University of Lomé Lome Togo; ^4^ Université Félix Houphouët‐Boigny Abidjan Côte d'Ivoire; ^5^ Centre Suisse de Recherches Scientifiques en Côte d'Ivoire (CSRS) Abidjan Côte d'Ivoire; ^6^ Laboratory of Applied Biology and Ecology, Faculty of Science University of Dschang Dschang Cameroon; ^7^ Transformation Pathways Department, Potsdam Institute for Climate Impact Research (PIK) Member of the Leibniz Association Potsdam Germany; ^8^ Re: Wild (Formerly Global Wildlife Conservation) Austin Texas USA; ^9^ Max Planck Institute for Evolutionary Anthropology Leipzig Germany; ^10^ Department of Biology, Centre for the Advanced Study of Collective Behaviour University of Konstanz Konstanz Germany; ^11^ Department of Animal Societies, Max Planck Institute of Animal Behaviour Konstanz Germany; ^12^ The Biodiversity Consultancy Cambridge UK; ^13^ Institute of Cognitive Science University of Osnabrück Osnabrück Germany; ^14^ German Centre for Integrative Biodiversity Research (iDiv) Halle‐Jena‐Leipzig Leipzig Germany; ^15^ Leipzig University Leipzig Germany; ^16^ The Faculty of ‘Artes Liberales’, University of Warsaw Warsaw Poland; ^17^ RSPB Centre for Conservation Science, The David Attenborough Building Cambridge UK; ^18^ Department of Anthropology University of Victoria Victoria British Columbia Canada; ^19^ Institute of Human Origins Arizona State University Tempe Arizona USA; ^20^ Animal Ecology and Tropical Biology, Biozentrum (Zoologie III) Wurzburg Germany; ^21^ Department of Primate Behavior and Evolution, Max Planck Institute for Evolutionary Anthropology Leipzig Germany; ^22^ Zooniverse Citizen Scientist, c/o Max Planck Institute for Evolutionary Anthropology Leipzig Germany; ^23^ Department of Anthropology University College London London UK; ^24^ Department of Human Origins, Max Planck Institute for Evolutionary Anthropology Leipzig Germany; ^25^ Greater Mahale Ecosystem Research and Conservation Project Busongola Tanzania; ^26^ Gashaka Primate Project Serti Nigeria; ^27^ German Primate Center—Leibniz Institute for Primate Research, Cognitive Ethology Laboratory Gottingen Germany; ^28^ Department of Anthropology Yale University New Haven Connecticut USA; ^29^ CNRS UMR 5229, Ape Social Mind Lab, Institute for Cognitive Sciences Bron Cedex France; ^30^ Tai Chimpanzee Project, Centre Suisse de Recherches Scientifiques en Côte d'Ivoire Abidjan Côte d'Ivoire; ^31^ International Institute Zittau Technische Universität Dresden Zittau Germany

**Keywords:** camera traps, mammal community composition, protected areas, threatened species, trophic guilds

## Abstract

The diversity and composition of mammal communities are strongly influenced by human activities, though these relationships may vary across broad scales. Understanding this variation is key to conservation, as it provides a baseline for planning and evaluating management interventions. We assessed variation in the structure and composition of Afrotropical medium and large mammal communities within and outside protected areas, and under varying human impact. We collected data at 512 locations from 22 study sites in 12 Afrotropical countries over 7 years and 3 months (2011–2018) with 164,474 camera trap days in total. Half of these sites are located inside protected areas and half in unprotected areas. The sites are comparable in that they all harbor at least one great ape species, indicating a minimum level of habitat similarity, though they experience varying degrees of human impact. We applied Bayesian Regression models to relate site protection status and the degree of human impact to mammal communities. Protected area status was positively associated with the proportion of all threatened species, independent of the degree of human impact. Similarly, species richness was associated with area protection but was more sensitive to human impact. For all other attributes of the mammal communities, the pattern was more complex. The influence of human impact partially overrides the positive effects of protected area status, resulting in comparable mammal communities being observed both within protected areas and in similarly remote locations outside these areas. We observed a common pattern for large carnivores, whose probability of occurrence declined significantly with increasing human impact, independent of site protection status. Mammal communities benefit from sustainability measures of socio‐economic context that minimize human impact. Our results support the notion that conservation of mammalian species can be achieved by reducing human impact through targeted conservation measures, adopting landscape‐level management strategies, fostering community engagement, and safeguarding remote habitats with high mammal diversity.

## Graphical abstract

Human activities significantly influence the diversity and community composition of Afrotropical mammal communities, varying across different scales and impacting conservation efforts. Our 7 year study using camera traps across 22 sites in 12 countries shows that while protected areas support higher species richness and more threatened species, human impact can override these benefits, particularly affecting large carnivores. Effective conservation requires addressing human impact through targeted measures, landscape‐level management, community engagement, and protecting remote habitats.

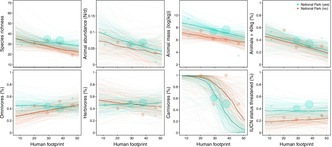

## 1 Introduction

The integrity and maintenance of tropical ecosystems worldwide is threatened by the substantial loss of wildlife (defaunation) due to various anthropogenic factors such as hunting and habitat conversion (Benítez‐López et al. 2019; De Paula Mateus et al. 2018; Venter et al. 2016; Young et al. 2016). This loss of wildlife is triggering ecological changes, such as the alteration of habitat dynamics (Rogers et al. 2021), the depletion of carbon storage stocks (Bello et al. 2015), and the disruption of natural ecosystem functions (Hoeks et al. 2020; Rogers et al. 2021). In some regions, it has also led to increased human–wildlife conflicts (Torres et al. 2018).

Defaunation disproportionately impacts megaherbivores and apex predators (Galetti et al. 2021; Ribeiro et al. 2016; Vale et al. 2021), resulting in reduced seed dispersal, altered vegetation patterns, disrupted nutrient cycling, and an imbalance in predator–prey dynamics across landscapes (Ripple et al. 2015). Despite these critical implications, there remains a limited understanding of the variations in composition and structure among Afrotropical mammal communities within and outside protected areas, especially under varying degrees of human impact.

Many factors have been shown to impact the distribution of medium to large mammal species such as habitat preference (Ayebare et al. 2018; Chabwela et al. 2017; Gorczynski et al. 2021), availability of resources [e.g., carnivorous species tend to be found in regions with high prey densities (Henschel et al. 2014; Torres‐Romero and Olalla‐Tárraga 2015)], human activities (Blom et al. 2005; Rija et al. 2020), and forest protection status (Geldmann et al. 2019). Compared to non‐protected forests, protected forest habitats have been shown to exhibit higher biodiversity and a greater number of threatened and endemic species (Jones et al. 2019). The long‐term survival of threatened large mammals—species that are sensitive to habitat fragmentation and often serve as indicators of broader ecosystem health—such as forest elephants ( *Loxodonta cyclotis* ) and great apes (family Hominidae), depends on the availability of vast, relatively undisturbed forest habitats (Anup 2017; Pacifici et al. 2020). Indeed, particularly remote and protected areas have proven to provide a crucial lifeline for many mammal species on the brink of extinction (Joppa et al. 2008; Schulze et al. 2018). Yet, though the provision of a status of formal protection for forests may aid the persistence of mammals and their habitats (Anup 2017; Pacifici et al. 2020), even protected areas are not void of anthropogenic impacts that alter the trophic structure and lower species richness (Geldmann et al. 2019; Rija et al. 2020). Nevertheless, in protected areas impacted by strong anthropogenic influence and lacking sufficient protection efforts, human pressures inevitably lead to altered trophic structure and lower species richness (Geldmann et al. 2019; Oberosler et al. 2020). By assessing changes in the composition and structure of mammal communities across different ecological conditions and protection statuses, we stand to gain valuable insights into the intertwined consequences of environmental changes and conservation strategies.

In our context, the ‘human footprint’ refers to a composite measure of various anthropogenic activities, such as habitat conversion, hunting and overexploitation of natural resources, that impact ecosystems (Benítez‐López et al. 2019; Ceballos et al. 2017; Junker et al. 2024; Laurance et al. 2012; Mu et al. 2022; Sanderson et al. 2002; Selier et al. 2015). These activities affect mammalian community composition, including species richness and diversity, the presence of threatened species, as well as the structural composition related to trophic guilds and body mass distribution (Brodie et al. 2015; Chillo and Ojeda 2012; Jones et al. 2019; Mu et al. 2022; Tucker et al. 2021; Vanthomme et al. 2013). The decline in animal (mammal) abundance and changes in community composition are strongly linked to altered herbivory patterns and prey dynamics, which in turn lead to changes in plant community composition, and reduced plant diversity (Atkins et al. 2019; Danell et al. 2006; Hoeks et al. 2020; Kays et al. 2010; Ripple et al. 2014). For example, the loss of mammalian seed dispersers can have a significant impact on forest regeneration, and thus the survival of numerous species of fruit‐bearing trees (Babweteera et al. 2007; Chapman and Dunham 2018; Cochrane 2003). Furthermore, in many forests the loss of keystone species, such as forest elephants, has induced a chain reaction: reduced seed dispersal by elephants has led to a diminished heterogeneity of forest structure and, eventually, decreased capacity for carbon storage (Poulsen et al. 2018).

Human pressures can disrupt the balance of trophic guilds within landscapes. For example, habitat destruction can reduce the availability of food resources for herbivores, leading to declines in their populations (Joppa et al. 2008). In turn, the decline of large herbivores can lead to the loss of heterotrophic biomass (Enquist et al. 2020), and the ensuing decline of carnivores, who are dependent on the herbivores, disrupt predator–prey dynamics and result in trophic cascades (Atkins et al. 2019; Hoeks et al. 2020). Unlike dietary specialists such as herbivores and carnivores, omnivores have more flexible diets. Therefore, they may benefit from having behavioral buffers to cope with human activities (Tucker et al. 2021). As a result, they may be less susceptible to disruptions in their food sources, environmental changes, and fluctuations in resource availability. To cope with ongoing habitat changes, some mammals may change their daily activity patterns in response to human presence (Gaynor et al. 2018; Nickel et al. 2020).

Camera traps are essential for assessing medium to large mammal community structure (Ahumada et al. 2011; Kays et al. 2010; Steenweg et al. 2017; Zlatanova and Popova 2018), enabling non‐intrusive sampling of diverse species, including those that are nocturnal, elusive and rare (O'Connell and Bailey 2011; Trolliet et al. 2014). The analysis of camera trap data yields valuable insights into spatial and temporal trends in faunal community metrics (Martin et al. 2015; Rovero et al. 2014). Large datasets, supported by community scientists (Amano et al. 2016; Barnard et al. 2017; Dickinson et al. 2010, 2012; Fraisl et al. 2022) or Artificial Intelligence (Fennell et al. 2022), allow researchers to address complex ecological and conservation questions (Arandjelovic et al. 2024; Hsing et al. 2022; Kays et al. 2015; Tagg et al. 2018). However, most African camera trap studies focus on local scales, and there is untapped potential to explore human‐driven variation in mammal communities across broader regions (Agha et al. 2018; Assou et al. 2021; Atkins et al. 2019; Djekda et al. 2020; Poulsen et al. 2018), especially regarding the combined effects of area protection status and human footprint on these communities.

Here, we use a large video camera trap dataset from the Pan African Programme: The Cultured Chimpanzee (PanAf) (Arandjelovic et al. 2024; Kühl et al. 2019) to assess the varying structure and composition of medium to large mammal communities under different site protection statuses, degrees, and contexts of human impact. We seek to better understand the association between species richness and diversity, abundance, mean body mass, trophic guilds, and threat status and the impact of the human footprint as well as site protection status (Mu et al. 2022; Venter et al. 2016). This investigation includes carnivores, which were analyzed separately due to their known sensitivity to human disturbance attributed to their home ranges (Ripple et al. 2014). While protected areas aim to support thriving mammalian communities, the level of human disturbance, due to illegal activities or weak enforcement, may undermine their effectiveness, which aligns with the broader discussion of human impact.

## 2 Methods

### 2.1 Study Area

**FIGURE 1:**
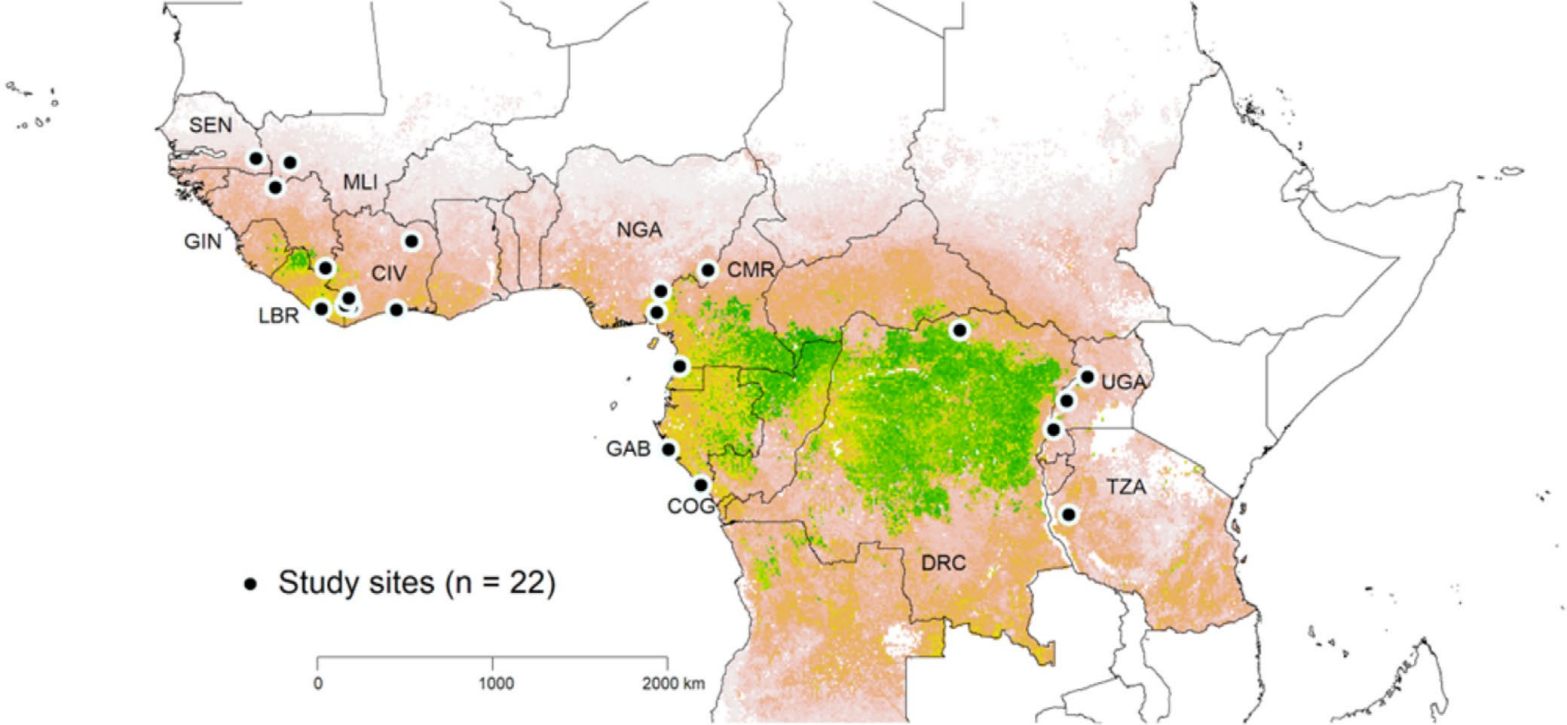
Location of PanAf research sites included in this study. The background represents estimated aboveground carbon density derived from Baccini et al. (2012). Greener colors indicate wetter forest‐dominated habitats and redder colors indicate drier, more open savanna or woodland habitats. Countries included in this study are labeled on the map following standard ISO alpha‐3 codes.

The data were collected at 512 camera locations in 22 African great ape habitat sites in 12 countries (Figure 1 and Table S1). The locations were composed of a range from predominantly forest or woodland to predominantly savanna vegetation, including transitional mosaic ecosystems. All locations had low to moderate human impact, with mean values for human footprint (see Table S1) ranging from 7 to 51 (up to 10 km outside the area), on a scale from 0 to 100 (Sanderson et al. 2002). We chose these sites to maximize the diversity of potential ecological conditions, potential variations in habitat characteristics, as well as variations in human disturbances that might affect the ecology of mammal communities. While environmental quality (e.g., habitat type, resources) is important, site protection status enforces reduced human impact, with human activities more effectively controlled under strict protection regimes. We have chosen various metrics to describe mammal communities (see below), to be able to compare relatively mammalian communities within protected areas to those outside of such areas under similar environmental conditions. Only sites in National Parks with IUCN categories I–II (Dudley 2008) were considered ‘protected’. All other sites were referred to as ‘unprotected’.

### 2.2 Camera Trapping

We acquired 7 years and 3 months (January 2011–April 2018) of camera trap records through the PanAf (http://panafrican.eva.mpg.de) project, which aims to describe wildlife diversity patterns and identify mechanisms that promote the development of behavioral diversity among populations of African great apes (Kalan et al. 2020). Camera traps were attached to trees at heights ranging from 0.90 to 1.1 m above ground level and oriented in the direction of animal trails, food trees, or bridges over the waterways, ensuring they had a sufficiently large field of view to capture the full‐body footage of animals (Arandjelovic et al. 2012). All camera traps (Bushnell Trophy Cam, various models) were set to record 1 min video when triggered. The resulting videos were annotated by a community of scientists on the Chimp&See platform (www.chimpandsee.org) and species were identified (Arandjelovic et al. 2024) hosted by Zooniverse (www.zooniverse.org). The number of observation days per site ranged from 129 to 2302 days (mean ± SD: 637 ± 437 days), with an average of 23.3 ± 8.2 (mean ± SD) camera traps per site. This resulted in a total of 744–10,519 camera trap days per site (mean ± SD: 7476 ± 2505), see Table S1.

We processed the data based on these final species identifications made by community scientists. Small mammal species (weighing less than ~1 kg) were excluded from the dataset, as they presented challenges in identification, unless they were likely to be detected by camera traps (Arandjelovic et al. 2024). We then assigned selected species traits, including average body mass, trophic guilds (omnivore, herbivore, carnivore), and threat status according to IUCN Red List of Threatened Species to each species (Kingdon 2015).

### 2.3 Data Analyses

To investigate the association between protected areas and human impact on medium and large‐bodied mammal communities, we used several Bayesian Regression Models (BRMs; Gelman et al. 2014), each including one of eight response variables (species richness, animal abundance per day, animal mass, percentage of larger mammals (weight > 40 kg), percentage of omnivores, percentage of herbivores, percentage of iucn status threatened, presence/absence of large carnivores), predictors (human footprint and protection status), and control variables (number of camera‐traps days) (Table S2).

For the response variables, we counted the number of animal detections per day per site. We were unable to establish a definitive time window to distinguish between independent detections reliably. Therefore, to minimize potential biases, we decided to count only one sighting per species per day. This approach aligns with standard practices in studies where defining independence between consecutive detections is challenging, particularly in cases of species with overlapping home ranges or high activity levels near camera traps (Burton et al. 2015). For simplicity, we refer in the following to this measure of daily richness as ‘abundance’. We used this variable as a proxy for animal abundance and subsequently refer to it as animal abundance per day. We additionally calculated the animal mass (log of the mean of the animal body masses based on daily sightings per species). Moreover, to understand potential changes in the mammal community structure due to site protection status and human disturbances, we calculated several metrics for each site. These included the percentage of larger mammals (weight > 40 kg), the percentage of omnivore species, the percentage of herbivore species of the total species richness in each case, a binomial variable accounting for the presence/absence of large carnivores (based on at least one sighting of a leopard *Panthera pardus* , lion *Panthera leo* , spotted hyena *Crocuta crocuta* , or African wild dog *Lycaon pictus* at a given site), and the percentage of species in the identified community considered globally threatened (combined IUCN statuses: “Critically Endangered”, “Endangered” and “Vulnerable”). The status of the study sites is a binary variable, with either being classified as National Park or no National Park. For simplicity, we refer in the following to it as either “protected” or “unprotected”.

The human footprint predictor was derived from a spatial composite layer integrating infrastructure, human population density, forest cover, and remoteness across 1‐km^2^‐grid cells with values ranging from 0 to 100 (e.g., Venter et al. 2016; Kennedy et al. 2019). For each camera location at a given site, we extracted the maximum human footprint within a 10 km radius and took the maximum of all values for a given site to capture the most intense human pressure within the site's surroundings. In the models, we included either one predictor variable to understand the effects independent of each other and additionally, we included an interaction term between the two (protection and human footprint, see model overview in Table S4) to estimate the effects of the predictors on each other.

Furthermore, to control for spatial autocorrelation, we included a Gaussian process over the longitude and latitude for each site by using the function *gp* from the R package ‘brms’ (Bürkner 2017). Finally, we controlled sampling effort by including the log of the sum of days that all cameras were deployed at each site as a covariate in all statistical models, in order to address variations in sampling effort across different locations (Table S2). Before running the model, we z‐transformed the continuous covariates to a mean of zero and a standard deviation of one to enable direct comparisons between estimates (Schielzeth 2010).

We fitted the models in R (R Core Team 2023) by using the function *brm* from the R package ‘brms’ (version 2.19.0). Depending on the response variable, we used either a Poisson (species richness), Gaussian (animal abundance per day, animal mass, % omnivores, % herbivores, % threatened species), or Bernoulli error distribution (carnivores) with the appropriate ‘log, identity or logit link’ function (Bürkner 2017). We used the package's default settings, which run 2000 iterations over four Markov Chain Monte Carlo (MCMC) methods, with a ‘warm‐up’ period of 1000 iterations per chain, for a total of 8000 usable posterior samples (Bürkner 2017). As we had no prior information on our effects, we used wide priors with a normal distribution, a mean of 0, and a standard deviation of 10 for the predictor variables. We visually inspected the results for any convergence issues, accordingly, we found that the MCMC showed stationarity and convergence to a common target, while Rhat values were all below 1.01, suggesting that different chains came to the same conclusion and that there were no divergent transitions after warm‐up (Gelman et al. 2014).

## 3 Results

### 3.1 Spatial Variation in Site‐Specific Mammalian Richness

We recorded 107 mammal species from 20 families across 22 sites, primarily medium to large‐sized (> 1 kg), with a few smaller species (< 1 kg) included due to their detectability by camera traps (Table S1). The species could be classified into three trophic guilds: 52 herbivores and frugivores, 35 omnivores and 20 carnivores. According to the IUCN Red List (IUCN 2023), 32 species are globally threatened, with 15 classified as ‘Vulnerable’, 13 ‘Endangered’, and four as ‘Critically Endangered’. Species rarefaction curves indicated that extended sampling periods would not have recorded many additional species at most sites except for the Azagny National Park (Azagny NP) in Côte d'Ivoire (Figure S7 and Tables S1 and S3). Overall, with a sampling effort of 637 ± 437 (mean ± SD) trap days (varying from 129 to 2302 days per site), we obtained 29.6 ± 8.2 (mean ± SD) species per site, ranging from four species at Azagny NP to 40 species at Korup NP, in Cameroon.

### 3.2 Biodiversity Indicators in Relation to Site Protection Status

**FIGURE 2:**
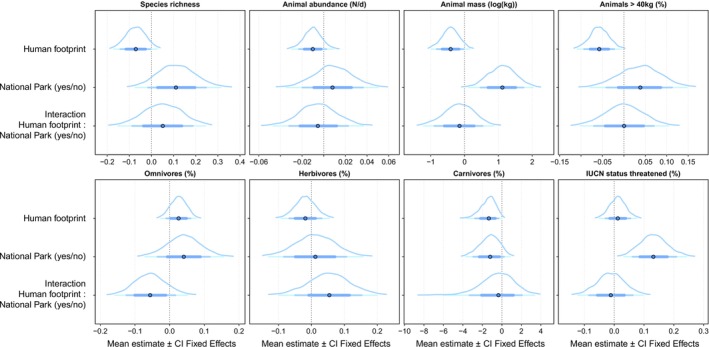
Posterior distribution and density of human footprint, site protection and the interaction between them on biodiversity variables. The plots show the estimates (dots; mean of the posterior distribution) and the 67%, 87%, and 97% credible intervals (blue bars). Additionally, the density of the posterior distribution is shown as a curved line above the horizontal credibility intervals.

**FIGURE 3:**
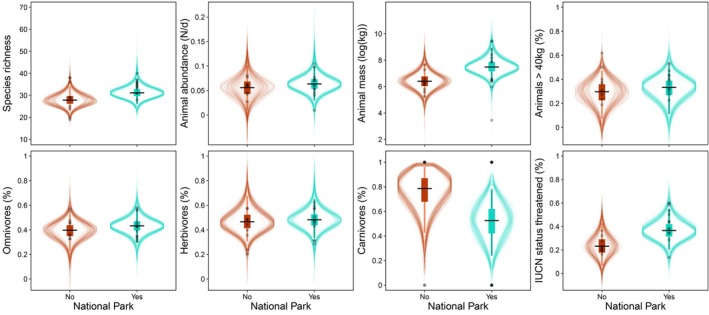
Posterior predictions of protection status on biodiversity variables represented as violin plots. The colored rectangles depict the 50% credibility intervals and the whiskers the 97% credibility intervals of the predicted posterior distribution (the horizontal black line depicts the mean). The lighter lines represent 150 draws from the posterior. Each dot represents a site and darker colors represent overlapping sites. Turquois colors represent protection, and red color represents unprotected areas.

We found a positive association between species richness and site protection status: posterior mean = 0.113, 95% BCI = −0.056–0.292 (Figure 2 and Table S4). Species richness was approximately 12% higher in protected sites compared to unprotected sites (estimates: 31.2 vs. 27.8 Figure 3 and Table S6). Animal abundance also showed differences between protected and unprotected sites: posterior mean = 0.008, 95% BCI = −0.029–0.045 (Figure 2 and Table S4). The means of daily animal abundance differed marginally by 14% between protected and unprotected sites (mean estimates: 0.063 vs. 0.056, Figure 3 and Table S6).

Animal mass was positively associated with site protection status while the percentage of larger animals showed only marginal differences between protected and unprotected areas [animal mass per day: posterior mean = 1.110 95% BCI = 0.245–1.954; − percentage of larger animals: posterior mean = 0.037 95% BCI = −0.066 to 0.137 (Figure 2, Table S4, and Figure S8a for distribution of weight classes)]. We found animal mass and the percentage of larger animals were marginally higher in protected sites vs. unprotected sites by 17% and 13%, respectively [mean estimates animal mass per day: 8.401 vs. 7.472; percentage of larger animals: 0.334 vs. 0.289, (Figure 3 and Table S6)].

The proportions of omnivores and herbivores recorded by camera traps showed only slight variations between protected and unprotected areas (omnivores: posterior mean = 0.041, 95% BCI = −0.062–0.139; herbivores: posterior mean = 0.012, 95% BCI = −0.114–0.138; Figure S6 and Table S4). The mean proportion of omnivores was 9% higher (0.433 vs. 0.397), and those of herbivores were 3% higher (mean estimates: 0.481 vs. 0.465) in protected sites compared to unprotected sites (Figure 3 and Table S6).

Our results suggest that the occurrence of large carnivores is higher in protected areas than in unprotected areas: posterior mean = −1.263 95% BCI = −3.458–0.630 (Figure 2 and Table S4). The occurrence of large carnivores was 33% higher in unprotected sites vs. protected sites (estimates: 0.786 vs. 0.526, Figure 3 and Table S6). The proportion of threatened species was positively related to site protection status: posterior mean = 0.133 95% BCI = 0.042–0.232 (Figure 2 and Table S4). On average, we found a proportion of 43% of threatened species in protected areas compared to 24% in unprotected areas, while the proportion of threatened species was higher by 56% in protected sites vs. unprotected sites (mean estimates: 0.366 vs. 0.234, Figure 3 and Table S6).

### 3.3 Effects of Human Footprint on Mammal Communities

**FIGURE 4:**
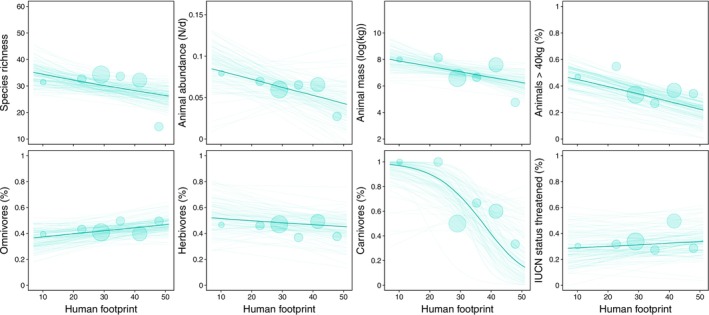
Posterior predictions for human footprint on biodiversity variables. The darker line represents the mean of the posterior distribution, and the lighter lines represent 150 draws from the posterior. The size of the circles indicates the sample size per value combination.

Species richness and daily abundance of animals are negatively associated with human footprint [species richness posterior mean =‐0.071 95% BCI = −0.161–0.017; daily animal abundance posterior mean =‐0.010 95% BCI = −0.028–0.007 (Figure 2 and Table S4)]. We found higher species richness in sites with low human footprint compared to sites with high human footprint by 25% (35 vs. 26, Figure 4 and Table S6), while daily animal abundance was also found noticeably higher by 51% for sites with high versus sites with low human footprint (mean estimates: 0.085 vs. 0.042, Figure 4 and Table S6).

Furthermore, we found a negative effect of human footprint on the estimated animal mass per site: posterior mean = −0.415 95% BCI =‐0.946‐0.088 (Figure 2 and Table S4), with a 22% difference between sites with low versus high human footprint (8.010 vs. 6.226, Figure 4 and Table S6). The percentage of larger animals in the communities is negatively associated with human footprint: posterior mean = −0.057 95% BCI =‐0.103‐0.011 (Figures 2 and 4, Figure S8b and Table S4, for distribution of weight classes) with a decrease of 53% between sites with low and high measures of human footprint (mean estimates: 0.463 versus 0.219, Figure 4 and Table S6).

The human footprint was positively associated with the proportion of omnivores and marginally negatively associated with the proportion of herbivores. Specifically, there was a 29% increase in the proportion of omnivores (posterior mean = 0.026, 95% BCI = −0.022 to 0.071), while the proportion of herbivores showed a decrease of −13.2% (posterior mean = −0.018, 95% BCI = −0.083 to 0.047; Figure 4 and Table S6). Additionally, the human footprint was negatively associated with the occurrence of large carnivores, with an 85% decrease in their presence observed between sites with low and high human footprint measures (Figures 2 and 4 and Table S6). No clear impact of the human footprint on threatened species has been detected, posterior mean (0.012, 95% BCI =‐0.043‐0.068; Figure 2 and Table S4).

### 3.4 Combined Effect of Site Protection Status and Human Footprint

**FIGURE 5:**
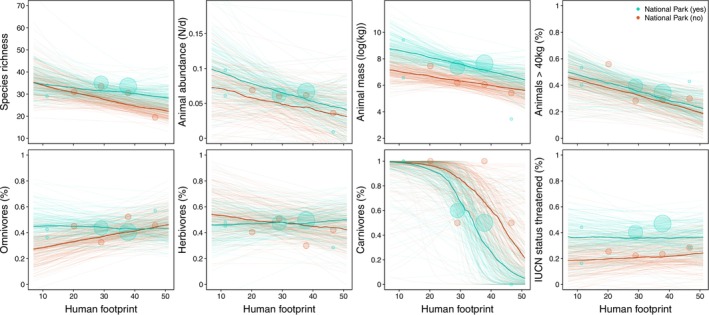
Posterior predictions for the interaction between protection status and the human footprint on biodiversity variables are represented by the continuous lines, where the darker one is the mean of the distribution, and the lighter lines represent 150 draws from the posterior. The size of the circles indicates the sample size per value combination.

For most mammal community attributes—such as species richness, daily animal abundance, animal mass, the proportion of large animals, and the occurrence of large carnivores—we found a negative relationship with the human footprint. This pattern was consistent across both protected and unprotected sites, although protected areas generally had higher baseline levels (intercepts). Specifically, species richness (posterior mean = −0.050, 95% BCI = −0.128 to 0.227), daily animal abundance (posterior mean = −0.005, 95% BCI = −0.043 to 0.031), animal mass (posterior mean = −0.160, 95% BCI = −1.119 to 0.748), the proportion of animals > 40 kg (posterior mean = 0.001, 95% BCI = −0.093 to 0.093), and the occurrence of large carnivores (posterior mean = −0.511, 95% BCI = −4.641 to 2.909) all declined with increasing human footprint (Figures 2 and 5 and Table S5).

Omnivores showed an increase in their proportion with higher human footprint in unprotected areas, while their proportion remained stable in protected areas (posterior mean = −0.056, 95% BCI = −0.147 to 0.039; Figures 2 and 5 and Table S5). In contrast, herbivores exhibited the opposite pattern: their proportion marginally decreased with higher human footprint in unprotected areas but remained stable in protected areas (posterior mean = −0.013, 95% BCI = −0.157 to 0.127; Figures 2 and 5 and Table S5).

As for the proportion of threatened species within the mammal communities, we found almost no effect of human footprint in both protected and unprotected areas. However, protected areas consistently showed higher baseline levels (intercepts) compared to unprotected areas (posterior mean = −0.011, 95% BCI = −0.108 to 0.086; Figures 2 and 5 and Table S5).

## 4 Discussion

Our study revealed a complex pattern of variation in mammal community structure and composition, influenced by both site protection status and human impact across the study sites. Camera trap data showed that protected areas did not consistently support more ‘intact’ mammal communities than unprotected areas with similar habitat conditions. This indicates that protection status alone does not sufficiently buffer mammal communities from human‐induced pressures, as both protected and unprotected areas exhibited similar patterns of change, differing primarily in their baseline conditions.

Evidence from our study indicates that human influence, rather than protection status, plays a more decisive role in shaping mammal community attributes. Weak enforcement of conservation policies, ineffective implementation of protective measures, and ongoing pressures such as hunting and illegal wildlife trade continue to undermine conservation efforts in the Afrotropical region (Allan et al. 2017; Amano et al. 2018; Dimobe et al. 2015; Furnell et al. 2015; Ripple et al. 2016). These results align with broader research indicating that current protection levels are insufficient to counteract human‐driven impacts on wildlife. Recognizing these limitations is crucial for refining conservation strategies and ensuring the long‐term persistence of mammal communities in human‐modified landscapes.

Our study reveals that human presence, as reflected by the human footprint, profoundly affects mammal communities, irrespective of site protection status. Sites with higher human footprints exhibited lower species richness, daily animal abundance, and biomass, with fewer large animals exceeding 40 kg in size. This emphasizes the importance of considering socio‐ecological linkages in conservation approaches, especially for species sensitive to human presence, such as great apes and large carnivores. These findings align with prior studies (e.g., Laurance et al. 2012), underscoring that protection alone may not sufficiently mitigate the negative effects of human activities on wildlife.

The decline in large carnivore populations was particularly striking, with an 85% reduction in their probability of occurrence in areas with intermediate human footprints compared to low‐impact sites. These results highlight the sensitivity of large carnivores to human disturbances, including habitat encroachment, prey depletion, and poaching (Ripple et al. 2014). Species such as lions, leopards, and hyenas are especially vulnerable, given their large home range requirements and direct conflicts with humans. Conservation strategies must prioritize habitat size, connectivity, and conflict mitigation to safeguard these apex predators.

Omnivores and herbivores, by contrast, displayed less sensitivity to human footprints, possibly due to their generalist and adaptive behaviors (Pineda‐Munoz and Alroy 2014). However, species‐specific responses within these groups likely vary, with certain herbivores or dietary specialists more affected by habitat alterations. These findings highlight the need for further research at the species level to uncover nuanced patterns of human impact.

Given the ongoing impact of human activities on wildlife and the potential for further expansion, it is crucial to enhance monitoring and strengthen the management of protected areas (Ma et al. 2024). While these areas serve as vital refuges for diverse species, including mammals, they remain susceptible to increasing anthropogenic pressures. Implementing effective management strategies to mitigate habitat degradation and poaching can help stabilize mammal communities over time, even in regions with significant human footprints (Geldmann et al. 2019; Watson et al. 2023). However, our study also highlights the vulnerability of unprotected areas, where human activities like habitat destruction and hunting are likely contributing to the near absence of mammals captured on camera traps. This emphasizes the complementary role of protected areas alongside broader conservation strategies that address the root causes of biodiversity loss.

Our findings indicate that site protection status alone is insufficient to shield mammals from human impacts, particularly in cases where management efforts fail to adequately address existing threats (e.g., Craigie et al. 2010). Furthermore, many protected areas were likely designated after human activities had already significantly altered local mammal communities (Hansen and DeFries 2007). Consequently, these areas may now preserve a compromised baseline rather than an untouched state. This is evidenced by the presence of threatened species within protected areas, indicating that these locations were often chosen to protect species already at risk. To prevent further biodiversity loss and restore ecological integrity, more proactive and rigorously enforced conservation measures are essential.

Our findings underscore the urgency of sustainable conservation strategies that address human‐driven impacts on mammal communities, both within and beyond protected areas. Protected areas play a vital role in providing safe environments for wildlife, but they must be complemented by broader strategies that address human activities such as habitat destruction, hunting, and land‐use changes (Barber et al. 2004). Ensuring the effectiveness of protected areas requires rigorous management practices, restoration of degraded habitats, and active mitigation of human‐wildlife conflicts.

Long‐term, localized, and time‐continuous studies are crucial for validating the space‐for‐time substitution approach used in this study and for disentangling the effects of protected area management, human footprint trajectories, and mammal community responses. Future research should explore site‐specific dynamics, particularly in areas like the Virungas, where mammal communities appear to have stabilized despite high human footprints (Hickey et al. 2019). This will help inform tailored conservation strategies for local conditions.

Additionally, conservation efforts should extend to remote forests, which still act as biodiversity hotspots, and focus on reconnecting fragmented habitats through landscape‐level management plans that involve local communities. Integrating socioeconomic factors into conservation planning is essential for ensuring practical and sustainable outcomes. Global frameworks like the Convention on Biological Diversity (CBD), the Aichi Biodiversity Targets, and the Kunming‐Montreal Global Biodiversity Framework highlight the importance of protected areas, management effectiveness, and community involvement in safeguarding biodiversity (Watson et al. 2023). By adopting a holistic approach that includes both protected and unprotected areas, addresses human impacts, and emphasizes community participation, we can create resilient landscapes capable of supporting diverse mammal communities in a rapidly changing Afrotropical region.

## Author Contributions

**Deogratias Tuyisingize:** conceptualization (equal), data curation (equal), formal analysis (equal), project administration (equal), writing – original draft (lead), writing – review and editing (lead). **Lars Kulik:** conceptualization (equal), data curation (equal), formal analysis (lead), writing – original draft (equal), writing – review and editing (equal). **Delagnon Assou:** data curation (equal), formal analysis (equal), writing – original draft (equal), writing – review and editing (equal). **Diorne Zausa:** data curation (equal), writing – original draft (equal), writing – review and editing (equal). **Solange Kamga:** data curation (equal), writing – original draft (equal). **Onella Mundi:** data curation (equal), writing – original draft (equal). **Stefanie Heinicke:** conceptualization (equal), funding acquisition (equal), writing – review and editing (equal). **Inza Kone:** conceptualization (equal), funding acquisition (equal), supervision (equal). **Samedi Jean Pierre Mucyo:** conceptualization (equal), writing – original draft (equal). **Colleen Stephens:** conceptualization (equal), data curation (equal), writing – review and editing (equal). **Tenekwetche Sop:** conceptualization (equal), funding acquisition (equal), supervision (equal). **Anthony Agbor:** conceptualization (equal), data curation (equal), methodology (equal), writing – review and editing (equal). **Samuel Angedakin:** conceptualization (equal), funding acquisition (equal), methodology (equal). **Emma Bailey:** conceptualization (equal), funding acquisition (equal), methodology (equal). **Mattia Bessone:** conceptualization (equal), methodology (equal). **Charlotte Coupland:** conceptualization (equal), methodology (equal), writing – review and editing (equal). **Josephine Head:** conceptualization (equal), methodology (equal), writing – original draft (equal). **Tobias Deschner:** conceptualization (equal), methodology (equal), writing – review and editing (equal). **Paula Dieguez:** conceptualization (equal), methodology (equal), writing – review and editing (equal). **Villard Ebot Egbe:** conceptualization (equal), methodology (equal), writing – review and editing (equal). **Anne‐Céline Granjon:** conceptualization (equal), methodology (equal), writing – review and editing (equal). **Thurston Cleveland Hicks:** conceptualization (equal), funding acquisition (equal), methodology (equal), writing – review and editing (equal). **Sorrel Jones:** conceptualization (equal), methodology (equal), writing – review and editing (equal). **Ammie K. Kalan:** conceptualization (equal), methodology (equal), writing – review and editing (equal). **Kevin E. Langergraber:** conceptualization (equal), methodology (equal), writing – review and editing (equal). **Juan Lapuente:** conceptualization (equal), project administration (equal), writing – review and editing (equal). **Kevin C. Lee:** conceptualization (equal), methodology (equal), writing – review and editing (equal). **Laura K. Lynn:** conceptualization (equal), methodology (equal), writing – review and editing (equal). **Nuria Maldonado:** conceptualization (equal), methodology (equal), writing – review and editing (equal). **Maureen S. McCarthy:** conceptualization (equal), methodology (equal), writing – review and editing (equal). **Amelia Meier:** conceptualization (equal), methodology (equal), writing – review and editing (equal). **Christophe Boesch:** conceptualization (equal), data curation (equal), funding acquisition (equal), project administration (equal), writing – original draft (equal). **Lucy Jayne Ormsby:** conceptualization (equal), methodology (equal), writing – review and editing (equal). **Alex K. Piel:** conceptualization (equal), methodology (equal), writing – review and editing (equal). **Lilah Sciaky:** conceptualization (equal), methodology (equal), writing – review and editing (equal). **Volker Sommer:** conceptualization (equal), methodology (equal), writing – review and editing (equal). **Fiona A. Stewart:** conceptualization (equal), writing – review and editing (equal). **Erin G. Wessling:** conceptualization (equal), methodology (equal), writing – review and editing (equal). **Jane Widness:** conceptualization (equal), methodology (equal), writing – review and editing (equal). **Roman M. Wittig:** conceptualization (equal), methodology (equal), writing – review and editing (equal). **Pauline Strohbach:** conceptualization (equal), data curation (equal), methodology (equal), resources (equal), writing – review and editing (equal). **Mimi Arandjelovic:** conceptualization (equal), funding acquisition (equal), methodology (equal), project administration (equal), resources (equal), writing – review and editing (equal). **Yntze van der Hoek:** formal analysis (equal), supervision (equal), writing – review and editing (equal). **Hjalmar S. Kühl:** conceptualization (lead), investigation (equal), methodology (equal), project administration (equal), resources (equal), supervision (lead), writing – review and editing (equal).

## Ethics Statement

Data collection used non‐invasive, remotely set camera traps, and hence did not involve direct contact or interaction with the animals. All data used in this manuscript were acquired from the Chimp&See platform and the PanAf consortium.

## Conflicts of Interest

The authors declare no conflicts of interest.

## Supporting information


Data S1.

Data S2.

## Data Availability

Data associated with this paper have been deposited in Dryad: https://doi.org/10.5061/dryad.r7sqv9sq3.
