# Supplementary material for: Complex Variation in Afrotropical Mammal Communities With Human Impact
Source: Ecol Evol. 2025 May 26;15(5):e71331. doi: 10.1002/ece3.71331 (PMC12104872; doi:10.1002/ece3.71331)
Supplement: Supplementary file 1 — Data S1. [file ECE3-15-e71331-s002.docx]

**Appendix Tables**

Appendix Table 1: Overview site information, sampling days, camera days, protection types, and variables (Species richness, animal abundance by day, animal mass by day, animal mass >40 kg, proportion of omnivores, proportion of herbivores, proportion of threatened species, presence and absence of large carnivores, human footprint) used across 12 countries.

| Site name | Country | Protection status of a given site | Year observation | N camera | Camera days | Animal abundance per day | Species richness | Presence of large carnivores | Percentage of larger mammals (> 40 kg) | Percentage of omnivores | Percentage of herbivores | Percentage of IUCN status threatened | Human footprint |
| --- | --- | --- | --- | --- | --- | --- | --- | --- | --- | --- | --- | --- | --- |
| Azagny | Ivory Coast | yes | 2012 | 12 | 744 | 0.009 | 5 | 0 | 0.429 | 0.571 | 0.286 | 0.286 | 45 |
| Bafing | Mali | no | 2014 | 33 | 9470 | 0.042 | 25 | 1 | 0.274 | 0.570 | 0.204 | 0.274 | 34 |
| Bakoun | Guinea | no | 2013 | 31 | 8063 | 0.081 | 36 | 1 | 0.311 | 0.475 | 0.397 | 0.197 | 34 |
| Bili | DRC | no | 2012 | 14 | 3063 | 0.061 | 24 | 1 | 0.618 | 0.548 | 0.237 | 0.323 | 23 |
| Budongo | Uganda | no | 2012 | 6 | 2672 | 0.045 | 20 | 1 | 0.364 | 0.455 | 0.488 | 0.207 | 46 |
| Bwindi | Uganda | yes | 2012 | 23 | 6954 | 0.105 | 35 | 1 | 0.472 | 0.579 | 0.312 | 0.378 | 40 |
| Campo Ma'an | Cameroon | yes | 2013 | 25 | 8087 | 0.070 | 37 | 0 | 0.390 | 0.462 | 0.467 | 0.344 | 32 |
| Conkouati | Congo | yes | 2014 | 28 | 10238 | 0.071 | 36 | 1 | 0.525 | 0.391 | 0.573 | 0.439 | 25 |
| Djouroutou | Ivory Coast | yes | 2012 | 23 | 6642 | 0.040 | 31 | 1 | 0.404 | 0.326 | 0.573 | 0.599 | 39 |
| East Nimba | Liberia | no | 2012 | 23 | 9672 | 0.027 | 19 | 0 | 0.235 | 0.458 | 0.356 | 0.364 | 51 |
| Gashaka | Nigeria | yes | 2012 | 18 | 7029 | 0.061 | 34 | 0 | 0.437 | 0.558 | 0.312 | 0.137 | 29 |
| GEPRENAF | Ivory Coast | no | 2013 | 36 | 6720 | 0.077 | 38 | 1 | 0.501 | 0.353 | 0.572 | 0.187 | 23 |
| Grebo Sala | Liberia | yes | 2012 | 26 | 9269 | 0.046 | 28 | 0 | 0.277 | 0.310 | 0.622 | 0.534 | 39 |
| Kayan | Senegal | no | 2012 | 41 | 9868 | 0.061 | 29 | 1 | 0.401 | 0.363 | 0.481 | 0.163 | 12 |
| Korup | Cameroon | yes | 2013 | 29 | 9824 | 0.073 | 40 | 0 | 0.226 | 0.444 | 0.508 | 0.344 | 34 |
| Loango | Gabon | yes | 2012 | 25 | 7381 | 0.098 | 34 | 1 | 0.533 | 0.425 | 0.451 | 0.442 | 7 |
| Mbe | Nigeria | no | 2013 | 13 | 10519 | 0.058 | 29 | 0 | 0.189 | 0.327 | 0.576 | 0.232 | 28 |
| Ngogo | Uganda | yes | 2012 | 20 | 7461 | 0.077 | 31 | 0 | 0.364 | 0.429 | 0.484 | 0.385 | 40 |
| Sapo | Liberia | yes | 2010 | 22 | 8037 | 0.053 | 33 | 1 | 0.298 | 0.350 | 0.594 | 0.597 | 32 |
| Tai-E | Ivory Coast | yes | 2013 | 24 | 6874 | 0.053 | 35 | 1 | 0.320 | 0.425 | 0.431 | 0.503 | 32 |
| Tai-R | Ivory Coast | yes | 2013 | 24 | 8228 | 0.060 | 36 | 1 | 0.318 | 0.342 | 0.472 | 0.581 | 39 |
| Issa Valley | Tanzania | no | 2011 | 16 | 7658 | 0.063 | 38 | 1 | 0.378 | 0.326 | 0.443 | 0.214 | 26 |

Appendix Table 2a: Overview of bayesian model structure and used variables

| Main effects | Response* ~ human footprint + protection status + sampling effort + spatAutocor |
| --- | --- |
| Main effects in Interaction | Response* ~ human footprint : protection status + sampling effort + spatAutocor |

* For the different response variables please see Appendix Table 2a.

Appendix Table 2b: A summary list of response, predictor, and control variables and how they were calculated.

| # | Variables | Type of variable | Definition |
| --- | --- | --- | --- |
| 1 | Species richness | Response | The number of distinct species recorded at each site. |
| 2 | Animal abundance per day | Response | The total number of species observed daily at each site (hereafter referred to as 'daily richness' for simplicity. |
| 3 | Animal mass | Response | The animal body masses based on daily sightings per species. |
| 4 | Percentage of larger mammals (weight > 40 kg) | Response | The proportion of mammal species at a given site with individual body masses exceeding 40 kg. |
| 5 | Percentage of omnivores | Response | The proportion of species of all species observed at a site that consume both plant and animal matter at a site. |
| 6 | Percentage of herbivores | Response | The proportion of species of all species observed at a site primarily consuming plant matter at a site. |
| 7 | Percentage of IUCN status threatened | Response | The proportion of animals classified as threatened by the IUCN Red List of Threatened Species, indicating declining populations or risk of extinction (vulnerable, endangered, critically endangered). |
| 8 | Presence/absence of large carnivores | Response | The occurrence or non-occurrence of large predatory species at a site |
| 9 | Human footprint | Predictor* | The extent of human activity and its impacts on species and habitats at a site (e.g., infrastructure, population density, deforestation). |
| 10 | Protection status of a given site | Predictor* | The designation of a site as protected follow IUCN categories. |
| 11 | Interaction between human footprint and protection status | Predictor** | Captures interaction between one variable and the other |
| 12 | Sampling effort (camera trap) | Control*** | The number of days camera traps were actively deployed at each site. |

- * For each response we ran one model comprising both predictors separately.
- ** Additionally, we ran for each response a model comprising the interaction term between the two predictors.
- *** The control was implemented in all the models.

Appendix Table 3: List of species observed, and their taxonomy considered in this study, along with the IUCN status of each species, their common names, and associated trophic guilds.

| # | Order | Family | Genus | Species | IUCN_status | Commun name | Dominant trophic guilds |
| --- | --- | --- | --- | --- | --- | --- | --- |
| 1 | Carnivora | Canidae | Canis | Canis adustus (Sundevall, 1847) | Least Concern | Side-striped Jackal | Omnivore |
| 2 | Carnivora | Canidae | Lycaon | Lycaon pictus (Temminck, 1820) | Endangered | African Wild Dog | Carnivore |
| 3 | Carnivora | Felidae | Caracal | Caracal aurata (Temminck, 1827) | Vulnerable | African Golden Cat | Carnivore |
| 4 | Carnivora | Felidae | Caracal | Caracal caracal (Schreber, 1776) | Least Concern | Caracal | Carnivore |
| 5 | Carnivora | Felidae | Leptailurus | Leptailurus serval (Schreber, 1776) | Least Concern | Serval | Carnivore |
| 6 | Carnivora | Felidae | Panthera | Panthera leo (Linnaeus, 1758) | Vulnerable | Lion | Carnivore |
| 7 | Carnivora | Felidae | Panthera | Panthera pardus (Linnaeus, 1758) | Vulnerable | Leopard | Carnivore |
| 8 | Carnivora | Herpestidae | Atilax | Atilax paludinosus (G.[Baron] Cuvier, 1829) | Least Concern | Marsh Mongoose | Omnivore |
| 9 | Carnivora | Herpestidae | Bdeogale | Bdeogale crassicauda (Peters, 1852) | Least Concern | Bushy-tailed Mongoose | Carnivore |
| 10 | Carnivora | Herpestidae | Bdeogale | Bdeogale nigripes (Pucheran, 1855) | Least Concern | Black-legged Mongoose | Carnivore |
| 11 | Carnivora | Herpestidae | Crossarchus | Crossarchus alexandri (Thomas & Wroughton, 1907) | Least Concern | Alexander's Cusimanse | Carnivore |
| 12 | Carnivora | Herpestidae | Crossarchus | Crossarchus obscurus (F.G. Cuvier, 1825) | Least Concern | Common Cusimanse | Carnivore |
| 13 | Carnivora | Herpestidae | Crossarchus | Crossarchus platycephalus (Goldman, 1984) | Least Concern | Flat-headed Cusimanse | Carnivore |
| 14 | Carnivora | Herpestidae | Herpestes | Herpestes sanguineus (Rüppell, 1835) | Least Concern | Slender Mongoose | Carnivore |
| 15 | Carnivora | Herpestidae | Ichneumia | Ichneumia albicauda (G. Cuvier, 1829) | Least Concern | White-tailed Mongoose | Carnivore |
| 16 | Carnivora | Herpestidae | Mungos | Mungos gambianus (Ogilby, 1835) | Least Concern | Gambian Mongoose | Carnivore |
| 17 | Carnivora | Herpestidae | Mungos | Mungos mungo (Gmelin, 1788) | Least Concern | Banded Mongoose | Omnivore |
| 18 | Carnivora | Hyaenidae | Crocuta | Crocuta crocuta (Erxleben, 1777) | Least Concern | Spotted Hyaena | Carnivore |
| 19 | Carnivora | Mustelidae | Aonyx | Aonyx capensis (Schinz, 1821) | Near Threatened | African Clawless Otter | Carnivore |
| 20 | Carnivora | Mustelidae | Mellivora | Mellivora capensis (Schreber, 1776) | Least Concern | Honey Badger | Carnivore |
| 21 | Carnivora | Nandiniidae | Nandinia | Nandinia binotata (Gray, 1830) | Least Concern | African Palm Civet | Frugivore |
| 22 | Carnivora | Viverridae | Civettictis | Civettictis civetta (Schreber, 1776) | Least Concern | Civet Civetta | Omnivore |
| 23 | Carnivora | Viverridae | Genetta | Genetta sp. | Vulnerable | Genet | Omnivore |
| 24 | Cetartiodactyla | Bovidae | Alcelaphus | Alcelaphus buselaphus (Pallas, 1766) | Least Concern | Hartebeest | Herbivore |
| 25 | Cetartiodactyla | Bovidae | Cephalophus | Cephalophus callipygus (Peters, 1876) | Least Concern | Peter's Duiker | Herbivore |
| 26 | Cetartiodactyla | Bovidae | Cephalophus | Cephalophus dorsalis (Gray, 1846) | Near Threatened | Bay Duiker | Herbivore |
| 27 | Cetartiodactyla | Bovidae | Cephalophus | Cephalophus jentinki (Thomas, 1892) | Endangered | Jentink's Duiker | Herbivore |
| 28 | Cetartiodactyla | Bovidae | Cephalophus | Cephalophus leucogaster (Gray, 1873) | Near Threatened | White-bellied Duiker | Omnivore |
| 29 | Cetartiodactyla | Bovidae | Cephalophus | Cephalophus niger (Gray, 1846) | Least Concern | Black Duiker | Herbivore |
| 30 | Cetartiodactyla | Bovidae | Cephalophus | Cephalophus nigrifrons (Gray, 1871) | Least Concern | Black-fronted Duiker | Herbivore |
| 31 | Cetartiodactyla | Bovidae | Cephalophus | Cephalophus ogilbyi (Waterhouse, 1838) | Least Concern | Ogilby's Duiker | Herbivore |
| 32 | Cetartiodactyla | Bovidae | Cephalophus | Cephalophus ogilbyi brookei (Thomas, 1903) | Vulnerable | Brooke's Duiker | Herbivore |
| 33 | Cetartiodactyla | Bovidae | Cephalophus | Cephalophus ogilbyi crusalbum | Near Threatened | White-legged Duiker | Herbivore |
| 34 | Cetartiodactyla | Bovidae | Cephalophus | Cephalophus rufilatus (Gray, 1846) | Least Concern | Red-flanked Duiker | Herbivore |
| 35 | Cetartiodactyla | Bovidae | Cephalophus | Cephalophus silvicultor (Afzelius, 1815) | Near Threatened | Yellow -backed Duiker | Omnivore |
| 36 | Cetartiodactyla | Bovidae | Cephalophus | Cephalophus weynsi (Thomas, 1901) | Least Concern | Weyns's Duiker | Herbivore |
| 37 | Cetartiodactyla | Bovidae | Cephalophus | Cephalophus zebra (Gray, 1838) | Vulnerable | Zebra Duiker | Herbivore |
| 38 | Cetartiodactyla | Bovidae | Hippotragus | Hippotragus equinus (É. Geoffroy Saint-Hilaire, 1803) | Least Concern | Roan Antelope | Herbivore |
| 39 | Cetartiodactyla | Bovidae | Kobus | Kobus ellipsiprymnus (Ogilbyi, 1833) | Least Concern | Waterbuck | Herbivore |
| 40 | Cetartiodactyla | Bovidae | Kobus | Kobus kob (Erxleben, 1777) | Least Concern | Kob | Herbivore |
| 41 | Cetartiodactyla | Bovidae | Madoqua | Madoqua sp. | Least Concern | Dik-Dik | Herbivore |
| 42 | Cetartiodactyla | Bovidae | Neotragus | Neotragus batesi (de Winton, 1903) | Least Concern | Bates' Pygmy Antelope | Herbivore |
| 43 | Cetartiodactyla | Bovidae | Neotragus | Neotragus pygmaeus (Linnaeus, 1758) | Least Concern | Royal Antelope | Herbivore |
| 44 | Cetartiodactyla | Bovidae | Ourebia | Ourebia ourebi (Zimmermann, 1783) | Least Concern | Oribi | Herbivore |
| 45 | Cetartiodactyla | Bovidae | Philantomba | Philantomba maxwellii (C.H. Smith, 1827) | Least Concern | Maxwell's Duiker | Herbivore |
| 46 | Cetartiodactyla | Bovidae | Philantomba | Philantomba monticola (Thunberg, 1789) | Least Concern | Blue Duiker | Herbivore |
| 47 | Cetartiodactyla | Bovidae | Philantomba | Philantomba sp. | Least Concern | Small Grey Duiker | Herbivore |
| 48 | Cetartiodactyla | Bovidae | Redunca | Redunca redunca (Pallas, 1767) | Least Concern | Reedbuck | Herbivore |
| 49 | Cetartiodactyla | Bovidae | Sylvicapra | Sylvicapra grimmia (Linnaeus, 1758) | Least Concern | Bush Duiker | Herbivore |
| 50 | Cetartiodactyla | Bovidae | Syncerus | Syncerus caffer nanus (Sparrman, 1779) | Near Threatened | Forest Buffalo | Herbivore |
| 51 | Cetartiodactyla | Bovidae | Tragelaphus | Tragelaphus eurycerus (Ogilbyi, 1837) | Near Threatened | Bongo | Herbivore |
| 52 | Cetartiodactyla | Bovidae | Tragelaphus | Tragelaphus scriptus (Pallas, 1766) | Least Concern | Bushbuck | Herbivore |
| 53 | Cetartiodactyla | Bovidae | Tragelaphus | Tragelaphus spekii (Speke, 1863) | Least Concern | Sitatunga | Herbivore |
| 54 | Cetartiodactyla | Hippopotamidae | Choeropsis | Choeropsis liberiensis (Morton, 1849) | Endangered | Pygmy Hippopotamus | Herbivore |
| 55 | Cetartiodactyla | Suidae | Hylochoerus | Hylochoerus meinertzhageni (Thomas, 1904) | Least Concern | Forest Hog | Omnivore |
| 56 | Cetartiodactyla | Suidae | Phacochoerus | Phacochoerus africanus (Gmelin, 1788) | Least Concern | Common Warthog | Herbivore |
| 57 | Cetartiodactyla | Suidae | Potamochoerus | Potamochoerus larvatus (F. Cuvier, 1822) | Least Concern | Bushpig | Omnivore |
| 58 | Cetartiodactyla | Suidae | Potamochoerus | Potamochoerus porcus (Linnaeus, 1758) | Least Concern | Red River Hog | Omnivore |
| 59 | Cetartiodactyla | Tragulidae | Hyemoschus | Hyemoschus aquaticus (Thomas, 1904) | Least Concern | Water Chevrotain | Omnivore |
| 60 | Pholidota | Manidae | Phataginus | Phataginus tricuspis (Rafinesque, 1821) | Endangered | White-bellied Pangolin | Carnivore |
| 61 | Pholidota | Manidae | Smutsia | Smutsia gigantea (Illiger, 1815) | Endangered | Giant Pangolin | Carnivore |
| 62 | Primates | Cercopithecidae | Allochrocebus | Allochrocebus lhoesti (P. Sclater, 1899) | Vulnerable | L'Hoest's Monkey | Omnivore |
| 63 | Primates | Cercopithecidae | Allochrocebus | Allochrocebus preussi (Matschie, 1898) | Endangered | Preuss's Monkey | Herbivore |
| 64 | Primates | Cercopithecidae | Allochrocebus | Allochrocebus solatus (Harrison, 1988) | Near Threatened | Sun-tailed Monkey | Omnivore |
| 65 | Primates | Cercopithecidae | Cercocebus | Cercocebus agilis (Milne-Edwards, 1886) | Least Concern | Agile Mangabey | Omnivore |
| 66 | Primates | Cercopithecidae | Cercocebus | Cercocebus atys (Audebert, 1797) | Vulnerable | Sooty Mangabey | Herbivore |
| 67 | Primates | Cercopithecidae | Cercocebus | Cercocebus lunulatus (Temminck, 1853) | Endangered | White-naped Mangabey | Herbivore |
| 68 | Primates | Cercopithecidae | Cercocebus | Cercocebus torquatus (Kerr, 1792) | Endangered | Red-capped Mangabey | Omnivore |
| 69 | Primates | Cercopithecidae | Cercopithecus | Cercopithecus ascanius (Audebert, 1799) | Least Concern | Red-tailed Monkey | Frugivore |
| 70 | Primates | Cercopithecidae | Cercopithecus | Cercopithecus campbelli (Waterhouse, 1838) | Near Threatened | Campbell's Monkey | Frugivore |
| 71 | Primates | Cercopithecidae | Cercopithecus | Cercopithecus cephus (Linnaeus, 1758) | Least Concern | Moustached Guenon | Omnivore |
| 72 | Primates | Cercopithecidae | Cercopithecus | Cercopithecus diana (Linnaeus, 1758) | Endangered | Diana Monkey | Omnivore |
| 73 | Primates | Cercopithecidae | Cercopithecus | Cercopithecus erythrotis (Waterhouse, 1838) | Vulnerable | Red-eared Monkey | Omnivore |
| 74 | Primates | Cercopithecidae | Cercopithecus | Cercopithecus lowei (Thomas, 1923) | Vulnerable | Lowe's Monkey | Frugivore |
| 75 | Primates | Cercopithecidae | Cercopithecus | Cercopithecus mitis (Wolf, 1822) | Least Concern | Blue Monkey | Omnivore |
| 76 | Primates | Cercopithecidae | Cercopithecus | Cercopithecus mona (Schreber, 1775) | Near Threatened | Mona Monkey | Omnivore |
| 77 | Primates | Cercopithecidae | Cercopithecus | Cercopithecus neglectus (Schlegel, 1876) | Least Concern | De Brazza's Monkey | Frugivore |
| 78 | Primates | Cercopithecidae | Cercopithecus | Cercopithecus petaurista (Schreber, 1774) | Near Threatened | Spot-nosed Monkey | Omnivore |
| 79 | Primates | Cercopithecidae | Cercopithecus | Cercopithecus pogonias (Bennett, 1833) | Near Threatened | Crowned Monkey | Frugivore |
| 80 | Primates | Cercopithecidae | Chlorocebus | Chlorocebus cynosuros (Scopoli, 1786) | Least Concern | Malbrouck Monkey | Omnivore |
| 81 | Primates | Cercopithecidae | Chlorocebus | Chlorocebus pygerythrus (F. Cuvier, 1821) | Least Concern | Vervet Monkey | Omnivore |
| 82 | Primates | Cercopithecidae | Chlorocebus | Chlorocebus sabaeus (Linnaeus, 1766) | Least Concern | Green Monkey | Omnivore |
| 83 | Primates | Cercopithecidae | Chlorocebus | Chlorocebus tantalus (Ogilby, 1841) | Least Concern | Tantalus Monkey | Omnivore |
| 84 | Primates | Cercopithecidae | Colobus | Colobus angolensis (P. Sclater, 1860) | Vulnerable | Angola Colobus | Herbivore |
| 85 | Primates | Cercopithecidae | Colobus | Colobus guereza (Rüppell, 1835) | Least Concern | Guereza | Herbivore |
| 86 | Primates | Cercopithecidae | Colobus | Colobus polykomos (I. Geoffroy Saint-Hilaire, 1834) | Endangered | King Colobus | Herbivore |
| 87 | Primates | Cercopithecidae | Colobus | Colobus satanas (Waterhouse, 1838) | Vulnerable | Black Colobus | Herbivore |
| 88 | Primates | Cercopithecidae | Erythrocebus | Erythrocebus patas (Schreber, 1774) | Near Threatened | Patas Monkey | Omnivore |
| 89 | Primates | Cercopithecidae | Lophocebus | Lophocebus albigena (Gray, 1850) | Vulnerable | Grey-cheeked Mangabey | Frugivore |
| 90 | Primates | Cercopithecidae | Mandrillus | Mandrillus leucophaeus (F. Cuvier, 1807) | Endangered | Drill | Omnivore |
| 91 | Primates | Cercopithecidae | Mandrillus | Mandrillus sphinx (Linnaeus, 1758) | Vulnerable | Mandrill | Omnivore |
| 92 | Primates | Cercopithecidae | Miopithecus | Miopithecus ogouensis (Kingdon, 1997) | Near Threatened | Gabon Talapoin | Omnivore |
| 93 | Primates | Cercopithecidae | Papio | Papio anubis (Lesson, 1827) | Least Concern | Olive Baboon | Omnivore |
| 94 | Primates | Cercopithecidae | Papio | Papio cynocephalus (Linnaeus, 1766) | Least Concern | Yellow Baboon | Omnivore |
| 95 | Primates | Cercopithecidae | Papio | Papio papio (Desmarest, 1820) | Near Threatened | Guinea Baboon | Omnivore |
| 96 | Primates | Cercopithecidae | Piliocolobus | Piliocolobus badius (Kerr, 1792) | Endangered | Western Red Colobus | Herbivore |
| 97 | Primates | Cercopithecidae | Piliocolobus | Piliocolobus sp. | Critically Endangered | Red Colobus | Herbivore |
| 98 | Primates | Cercopithecidae | Procolobus | Procolobus verus (Van Beneden, 1838) | Vulnerable | Olive Colobus | Herbivore |
| 99 | Primates | Hominidae | Gorilla | Gorilla beringei (Matschie, 1903) | Critically Endangered | Eastern Gorilla | Herbivore |
| 100 | Primates | Hominidae | Gorilla | Gorilla gorilla (Savage, 1847) | Critically Endangered | Western Gorilla | Frugivore |
| 101 | Primates | Hominidae | Pan | Pan troglodytes (Blumenbach, 1799) | Endangered | Chimp | Omnivore |
| 102 | Proboscidea | Elephantidae | Loxodonta | Loxodonta cyclotis (Matschie, 1900) | Critically Endangered | African Forest Elephant | Herbivore |
| 103 | Rodentia | Hystricidae | Atherurus | Atherurus africanus (Gray, 1842) | Least Concern | African Brush-tailed Porcupine | Omnivore |
| 104 | Rodentia | Nesomyidae | Cricetomys | Cricetomys sp. | Least Concern | Pouched Rat | Omnivore |
| 105 | Rodentia | Sciuridae | Protoxerus | Protoxerus aubinnii (Gray, 1873) | Near Threatened | Slender-tailed Squirrel | Frugivore |
| 106 | Rodentia | Thryonomyidae | Thryonomys | Thryonomys swinderianus (Temminck, 1827) | Least Concern | Cane Rat | Herbivore |
| 107 | Tubulidentata | Orycteropodidae | Orycteropus | Orycteropus afer (Pallas, 1766) | Least Concern | Aardvark | Carnivore |

Appendix Table 4: Model estimates for protection status and human footprint on species richness, daily animal abundance, animal mass daily ratio, and the percentages of species with a body mass > 40 kg, considered as globally threatened, or classified as omnivore, herbivore, or carnivore.

| Response | variables | Estimate | Est.Error | l.95..CI | u.95..CI | Rhat | Bulk_ESS | Tail_ESS |
| --- | --- | --- | --- | --- | --- | --- | --- | --- |
| Animal richness | Intercept | 3.383 | 0.107 | 3.167 | 3.566 | 1.003 | 2064 | 1590 |
| Animal richness | human footprint | -0.071 | 0.046 | -0.161 | 0.017 | 1.001 | 4407 | 2999 |
| Animal richness | observation effort | 0.273 | 0.062 | 0.158 | 0.398 | 1 | 4832 | 2910 |
| Animal richness | Intercept | 3.338 | 0.113 | 3.128 | 3.567 | 1.001 | 1979 | 1680 |
| Animal richness | protection (yes) | 0.113 | 0.089 | -0.056 | 0.292 | 1.001 | 3516 | 2550 |
| Animal richness | observation effort | 0.284 | 0.06 | 0.171 | 0.406 | 1 | 3916 | 2651 |
| Animal abundance daily mean | Intercept | 0.062 | 0.09 | -0.116 | 0.248 | 1.002 | 1819 | 2041 |
| Animal abundance daily mean | human footprint | -0.01 | 0.009 | -0.028 | 0.007 | 1.001 | 4214 | 2317 |
| Animal abundance daily mean | Intercept | 0.057 | 0.082 | -0.109 | 0.222 | 1.001 | 1839 | 2007 |
| Animal abundance daily mean | protection (yes) | 0.008 | 0.019 | -0.029 | 0.045 | 1.002 | 3404 | 2607 |
| log of mean animal mass | Intercept | 6.943 | 0.333 | 6.289 | 7.599 | 1.001 | 2483 | 1964 |
| log of mean animal mass | human footprint | -0.415 | 0.262 | -0.946 | 0.088 | 1.001 | 2767 | 2831 |
| log of mean animal mass | observation effort | 0.669 | 0.258 | 0.157 | 1.178 | 1.001 | 2883 | 2091 |
| log of mean animal mass | Intercept | 6.45 | 0.478 | 5.527 | 7.353 | 1.001 | 1806 | 1944 |
| log of mean animal mass | protection (yes) | 1.11 | 0.431 | 0.245 | 1.954 | 1.001 | 3511 | 2113 |
| log of mean animal mass | observation effort | 0.849 | 0.23 | 0.377 | 1.281 | 1.001 | 1343 | 2283 |
| Proportion of weightclass 4 | Intercept | 0.389 | 0.074 | 0.252 | 0.535 | 1.004 | 1263 | 1252 |
| Proportion of weightclass 4 | human footprint | -0.057 | 0.023 | -0.103 | -0.011 | 1 | 3096 | 3063 |
| Proportion of weightclass 4 | observation effort | -0.044 | 0.022 | -0.088 | -0.001 | 1 | 2311 | 3189 |
| Proportion of weightclass 4 | Intercept | 0.394 | 0.073 | 0.25 | 0.543 | 1 | 1813 | 2001 |
| Proportion of weightclass 4 | protection (yes) | 0.037 | 0.051 | -0.066 | 0.137 | 1 | 3211 | 3329 |
| Proportion of weightclass 4 | observation effort | -0.027 | 0.024 | -0.075 | 0.02 | 1.001 | 2315 | 2831 |
| Proportion of omnivores | Intercept | 0.439 | 0.071 | 0.286 | 0.578 | 1.001 | 1649 | 1331 |
| Proportion of omnivores | human footprint | 0.026 | 0.024 | -0.022 | 0.071 | 1.002 | 2618 | 2237 |
| Proportion of omnivores | observation effort | -0.043 | 0.022 | -0.088 | 0 | 1.006 | 3716 | 2908 |
| Proportion of omnivores | Intercept | 0.413 | 0.091 | 0.239 | 0.581 | 1.003 | 1228 | 1069 |
| Proportion of omnivores | protection (yes) | 0.041 | 0.051 | -0.062 | 0.139 | 1.001 | 2356 | 2609 |
| Proportion of omnivores | observation effort | -0.05 | 0.023 | -0.095 | -0.005 | 1 | 2661 | 2500 |
| Proportion of herbivores | Intercept | 0.434 | 0.077 | 0.271 | 0.585 | 1 | 2198 | 2230 |
| Proportion of herbivores | human footprint | -0.018 | 0.033 | -0.083 | 0.047 | 1.002 | 3212 | 2941 |
| Proportion of herbivores | observation effort | 0.053 | 0.029 | -0.007 | 0.107 | 1.001 | 3539 | 3052 |
| Proportion of herbivores | Intercept | 0.433 | 0.081 | 0.27 | 0.596 | 1.001 | 2285 | 2186 |
| Proportion of herbivores | protection (yes) | 0.012 | 0.064 | -0.114 | 0.138 | 1.001 | 3309 | 2923 |
| Proportion of herbivores | observation effort | 0.055 | 0.03 | -0.004 | 0.114 | 1.001 | 3806 | 2795 |
| Occurrence of large carnivores (yes/no) | Intercept | 0.798 | 0.586 | -0.291 | 1.991 | 1.006 | 4209 | 2579 |
| Occurrence of large carnivores (yes/no) | human footprint | -1.444 | 0.848 | -3.293 | -0.087 | 1.001 | 3455 | 2031 |
| Occurrence of large carnivores (yes/no) | observation effort | -0.238 | 0.603 | -1.446 | 0.931 | 1.001 | 5415 | 3083 |
| Occurrence of large carnivores (yes/no) | Intercept | 1.424 | 0.871 | -0.132 | 3.279 | 1.001 | 4392 | 2582 |
| Occurrence of large carnivores (yes/no) | protection (yes) | -1.263 | 1.056 | -3.458 | 0.63 | 1.001 | 5273 | 2982 |
| Occurrence of large carnivores (yes/no) | observation effort | 0.116 | 0.568 | -1.057 | 1.249 | 1.003 | 5528 | 2502 |
| Proportion of IUCN_status threatened | Intercept | 0.304 | 0.1 | 0.075 | 0.5 | 1.006 | 1490 | 1605 |
| Proportion of IUCN_status threatened | human footprint | 0.012 | 0.028 | -0.043 | 0.068 | 1.001 | 3000 | 2731 |
| Proportion of IUCN_status threatened | observation effort | 0.053 | 0.027 | 0.001 | 0.104 | 1.001 | 2679 | 2782 |
| Proportion of IUCN_status threatened | Intercept | 0.242 | 0.08 | 0.079 | 0.399 | 1.002 | 1651 | 1671 |
| Proportion of IUCN_status threatened | protection (yes) | 0.133 | 0.048 | 0.042 | 0.232 | 1.001 | 2687 | 2590 |
| Proportion of IUCN_status threatened | observation effort | 0.048 | 0.022 | 0.003 | 0.091 | 1.001 | 2827 | 3231 |

Appendix Table 5: Model estimates for the interaction between protection status and the human footprint on species richness, daily animal abundance, animal mass daily ratio, the ratio of animals greater than 40 kg, ratio of omnivores, the ratio of herbivores, the occurrence of large carnivores, the ratio of species with IUCN status threatened.

|  | response | variables | Estimate | Est.Error | l.95..CI | u.95..CI | Rhat | Bulk_ESS | Tail_ESS |
| --- | --- | --- | --- | --- | --- | --- | --- | --- | --- |
| 1 | Animal richness | Intercept | 3.301 | 0.112 | 3.081 | 3.526 | 1 | 2568 | 2519 |
| 2 | Animal richness | protection (yes) | 0.142 | 0.09 | -0.029 | 0.325 | 1 | 4055 | 2993 |
| 3 | Animal richness | human footprint | -0.106 | 0.062 | -0.229 | 0.016 | 1 | 3780 | 2918 |
| 4 | Animal richness | observation effort | 0.274 | 0.063 | 0.157 | 0.399 | 1 | 4636 | 2908 |
| 5 | Animal richness | protection (yes) * human footprint | 0.05 | 0.091 | -0.128 | 0.227 | 1 | 3403 | 2754 |
| 6 | Animal abundance daily mean | Intercept | 0.057 | 0.091 | -0.137 | 0.246 | 1 | 2145 | 1665 |
| 7 | Animal abundance daily mean | protection (yes) | 0.014 | 0.021 | -0.027 | 0.056 | 1 | 4060 | 2782 |
| 8 | Animal abundance daily mean | human footprint | -0.009 | 0.012 | -0.033 | 0.015 | 1 | 3841 | 2154 |
| 9 | Animal abundance daily mean | protection (yes) * human footprint | -0.005 | 0.018 | -0.043 | 0.031 | 1 | 3664 | 2623 |
| 10 | log of mean animal mass | Intercept | 6.303 | 0.409 | 5.517 | 7.135 | 1 | 3256 | 2733 |
| 11 | log of mean animal mass | protection (yes) | 1.175 | 0.441 | 0.321 | 2.041 | 1 | 4998 | 2850 |
| 12 | log of mean animal mass | human footprint | -0.406 | 0.298 | -1.009 | 0.172 | 1 | 3354 | 2633 |
| 13 | log of mean animal mass | observation effort | 0.655 | 0.241 | 0.156 | 1.123 | 1 | 1866 | 2594 |
| 14 | log of mean animal mass | protection (yes) * human footprint | -0.16 | 0.469 | -1.119 | 0.748 | 1 | 2164 | 2348 |
| 15 | Proportion of weightclass 4 | Intercept | 0.365 | 0.077 | 0.213 | 0.52 | 1 | 1560 | 1575 |
| 16 | Proportion of weightclass 4 | protection (yes) | 0.04 | 0.05 | -0.059 | 0.14 | 1 | 3223 | 2816 |
| 17 | Proportion of weightclass 4 | human footprint | -0.06 | 0.03 | -0.119 | 0.001 | 1 | 2749 | 2374 |
| 18 | Proportion of weightclass 4 | observation effort | -0.045 | 0.024 | -0.093 | 0.002 | 1 | 2806 | 2844 |
| 19 | Proportion of weightclass 4 | protection (yes) * human footprint | 0.001 | 0.047 | -0.093 | 0.093 | 1 | 2827 | 2655 |
| 20 | Proportion of omnivores | Intercept | 0.406 | 0.09 | 0.207 | 0.578 | 1 | 1645 | 1996 |
| 21 | Proportion of omnivores | protection (yes) | 0.062 | 0.054 | -0.049 | 0.165 | 1 | 1801 | 2466 |
| 22 | Proportion of omnivores | human footprint | 0.049 | 0.031 | -0.015 | 0.111 | 1 | 1567 | 1714 |
| 23 | Proportion of omnivores | observation effort | -0.05 | 0.021 | -0.092 | -0.006 | 1 | 3110 | 2823 |
| 24 | Proportion of omnivores | protection (yes) * human footprint | -0.056 | 0.047 | -0.147 | 0.039 | 1 | 1764 | 2589 |
| 25 | Proportion of herbivores | Intercept | 0.439 | 0.098 | 0.25 | 0.643 | 1 | 1550 | 1330 |
| 26 | Proportion of herbivores | protection (yes) | -0.013 | 0.074 | -0.157 | 0.127 | 1 | 1417 | 2537 |
| 27 | Proportion of herbivores | human footprint | -0.04 | 0.043 | -0.124 | 0.047 | 1 | 1475 | 2473 |
| 28 | Proportion of herbivores | observation effort | 0.056 | 0.03 | -0.007 | 0.114 | 1 | 3657 | 2948 |
| 29 | Proportion of herbivores | protection (yes) * human footprint | 0.054 | 0.067 | -0.079 | 0.185 | 1 | 1656 | 2460 |
| 30 | Occurrence of large carnivores (yes/no) | Intercept | 1.784 | 1.27 | -0.398 | 4.352 | 1 | 2820 | 1433 |
| 31 | Occurrence of large carnivores (yes/no) | protection (yes) | -1.203 | 1.567 | -4.244 | 1.554 | 1 | 2611 | 1103 |
| 32 | Occurrence of large carnivores (yes/no) | human footprint | -1.616 | 1.334 | -4.5 | 0.396 | 1.01 | 1166 | 510 |
| 33 | Occurrence of large carnivores (yes/no) | observation effort | -0.312 | 0.695 | -1.735 | 1.015 | 1 | 3524 | 1792 |
| 34 | Occurrence of large carnivores (yes/no) | protection (yes) * human footprint | -0.511 | 2.024 | -4.641 | 2.909 | 1.01 | 1267 | 377 |
| 35 | Proportion of IUCN_status threatened | Intercept | 0.247 | 0.078 | 0.083 | 0.407 | 1 | 1446 | 1779 |
| 36 | Proportion of IUCN_status threatened | protection (yes) | 0.139 | 0.055 | 0.033 | 0.253 | 1 | 2296 | 2204 |
| 37 | Proportion of IUCN_status threatened | human footprint | 0.013 | 0.03 | -0.05 | 0.072 | 1 | 2833 | 2607 |
| 38 | Proportion of IUCN_status threatened | observation effort | 0.049 | 0.024 | 0 | 0.095 | 1 | 2919 | 2647 |
| 39 | Proportion of IUCN_status threatened | protection (yes) * human footprint | -0.011 | 0.049 | -0.108 | 0.086 | 1 | 2646 | 2704 |

Appendix Table 6: Differences in predictions for models with protection status and the human footprint on species richness, daily animal abundance, animal mass daily ratio, the ratio of animals greater than 40 kg, ratio of omnivores, the ratio of herbivores, the occurrence of large carnivores, the ratio of species with IUCN status threatened.

|  | response | variables | Level | est | SE | low | upp | Change_% |
| --- | --- | --- | --- | --- | --- | --- | --- | --- |
| 1 | Animal richness | Human footprint | 1 | 35.142 | 4.456 | 31.332 | 39.758 | -25.4 |
| 2 | Animal richness | Human footprint | 100 | 26.2 | 3.036 | 23.363 | 29.23 |  |
| 3 | Animal richness | National Park (yes/no) | no | 27.841 | 2.601 | 25.554 | 30.454 | 12 |
| 4 | Animal richness | National Park (yes/no) | yes | 31.175 | 2.39 | 29.01 | 33.557 |  |
| 5 | Animal abundance daily mean | Human footprint | 1 | 0.085 | 0.022 | 0.065 | 0.105 | -50.6 |
| 6 | Animal abundance daily mean | Human footprint | 100 | 0.042 | 0.019 | 0.024 | 0.061 |  |
| 7 | Animal abundance daily mean | National Park (yes/no) | no | 0.056 | 0.019 | 0.037 | 0.074 | 13.9 |
| 8 | Animal abundance daily mean | National Park (yes/no) | yes | 0.063 | 0.015 | 0.05 | 0.078 |  |
| 9 | log of mean animal mass | Human footprint | 1 | 8.01 | 0.691 | 7.324 | 8.624 | -22.3 |
| 10 | log of mean animal mass | Human footprint | 100 | 6.226 | 0.613 | 5.654 | 6.791 |  |
| 11 | log of mean animal mass | National Park (yes/no) | no | 6.401 | 0.54 | 5.876 | 6.91 | 16.7 |
| 12 | log of mean animal mass | National Park (yes/no) | yes | 7.472 | 0.508 | 7.014 | 8.014 |  |
| 13 | Proportion of weightclass 4 | Human footprint | 1 | 0.463 | 0.088 | 0.371 | 0.539 | -52.8 |
| 14 | Proportion of weightclass 4 | Human footprint | 100 | 0.219 | 0.076 | 0.143 | 0.287 |  |
| 15 | Proportion of weightclass 4 | National Park (yes/no) | no | 0.296 | 0.1 | 0.196 | 0.383 | 12.7 |
| 16 | Proportion of weightclass 4 | National Park (yes/no) | yes | 0.334 | 0.09 | 0.239 | 0.411 |  |
| 17 | Proportion of omnivores | Human footprint | 1 | 0.365 | 0.076 | 0.289 | 0.434 | 28.6 |
| 18 | Proportion of omnivores | Human footprint | 100 | 0.469 | 0.07 | 0.403 | 0.535 |  |
| 19 | Proportion of omnivores | National Park (yes/no) | no | 0.397 | 0.065 | 0.329 | 0.456 | 8.9 |
| 20 | Proportion of omnivores | National Park (yes/no) | yes | 0.433 | 0.053 | 0.379 | 0.485 |  |
| 21 | Proportion of herbivores | Human footprint | 1 | 0.52 | 0.108 | 0.424 | 0.625 | -13.2 |
| 22 | Proportion of herbivores | Human footprint | 100 | 0.451 | 0.094 | 0.359 | 0.536 |  |
| 23 | Proportion of herbivores | National Park (yes/no) | no | 0.465 | 0.081 | 0.391 | 0.548 | 3.4 |
| 24 | Proportion of herbivores | National Park (yes/no) | yes | 0.481 | 0.067 | 0.42 | 0.546 |  |
| 25 | Occurrence of large carnivores (yes/no) | Human footprint | 1 | 0.979 | 0.028 | 0.89 | 0.998 | -85.1 |
| 26 | Occurrence of large carnivores (yes/no) | Human footprint | 100 | 0.146 | 0.151 | 0.04 | 0.357 |  |
| 27 | Occurrence of large carnivores (yes/no) | National Park (yes/no) | no | 0.786 | 0.138 | 0.634 | 0.896 | -33.1 |
| 28 | Occurrence of large carnivores (yes/no) | National Park (yes/no) | yes | 0.526 | 0.147 | 0.383 | 0.659 |  |
| 29 | Proportion of IUCN_status threatened | Human footprint | 1 | 0.285 | 0.11 | 0.18 | 0.392 | 19 |
| 30 | Proportion of IUCN_status threatened | Human footprint | 100 | 0.339 | 0.11 | 0.236 | 0.44 |  |
| 31 | Proportion of IUCN_status threatened | National Park (yes/no) | no | 0.234 | 0.085 | 0.154 | 0.317 | 56.5 |
| 32 | Proportion of IUCN_status threatened | National Park (yes/no) | yes | 0.366 | 0.079 | 0.293 | 0.446 |  |
