# Supplementary material for: Complex Variation in Afrotropical Mammal Communities With Human Impact
Source: Ecol Evol. 2025 May 26;15(5):e71331. doi: 10.1002/ece3.71331 (PMC12104872; doi:10.1002/ece3.71331)
Supplement: Supplementary file 2 — Data S2. [file ECE3-15-e71331-s001.docx]

**Supplementary Figures**

**
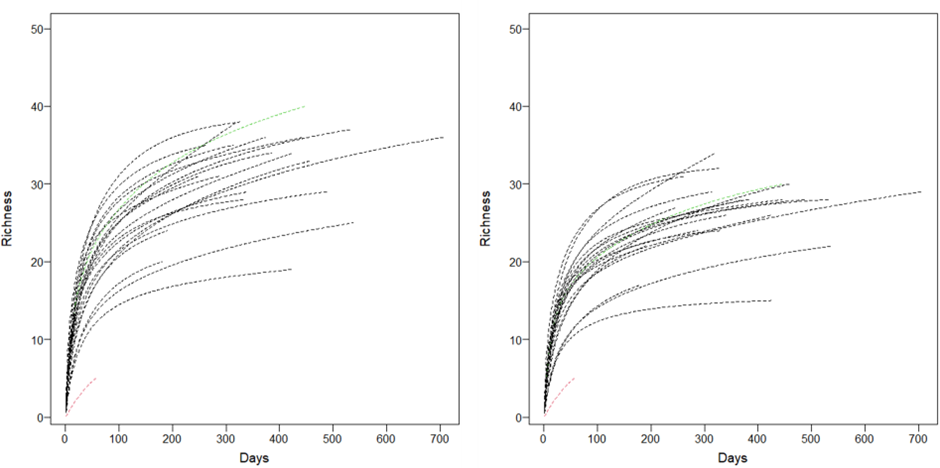
**

**Figure 6:** Species rarefaction curves. The lines represent the rarefaction curve for each of the 22 sites. The figure on the left includes arboreal species, and the one on the right does not. The red line indicates the Azagny National Park (lowest richness) and the green line is the Korup National Park (highest richness in this dataset).

**
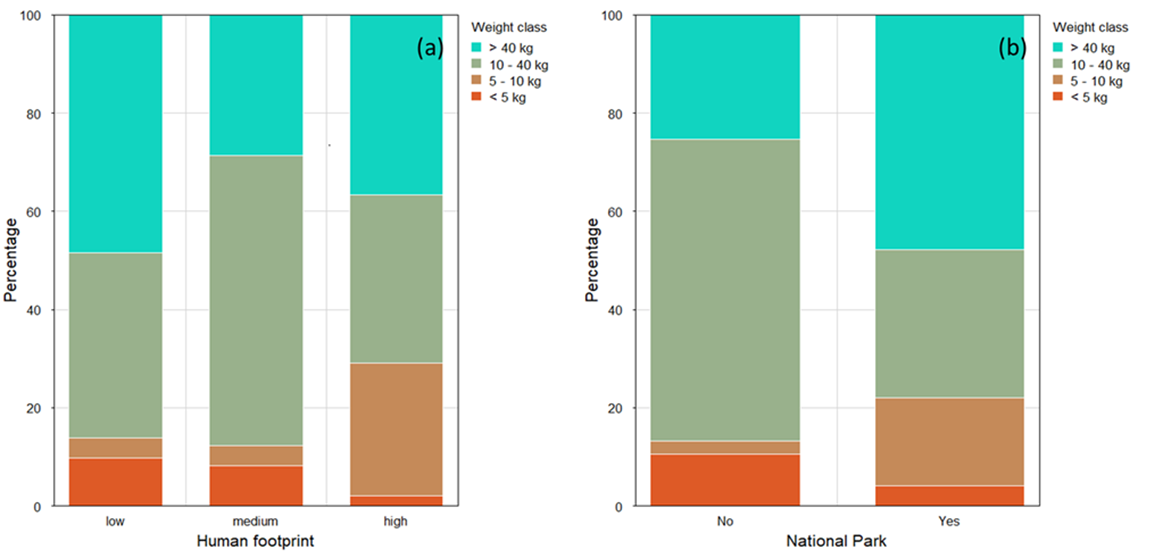
**

**Figure 7:** Percentage of mammal weight classes by human footprint level (a) and site protection (b)


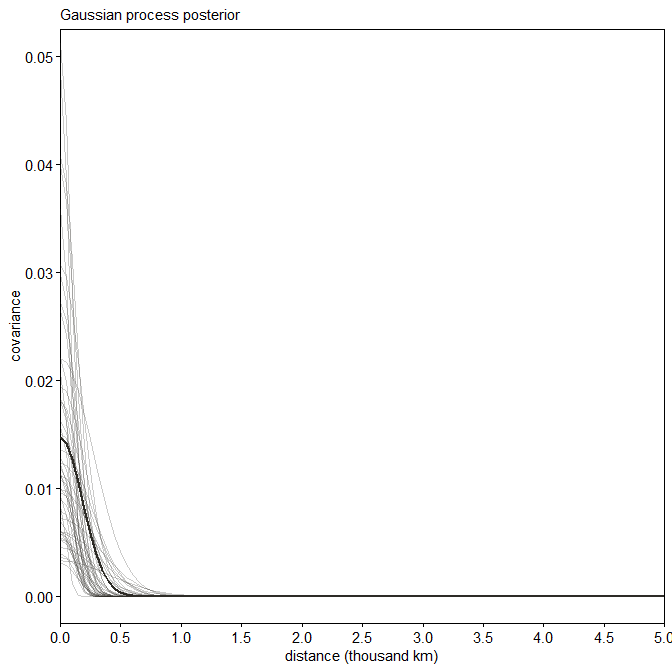

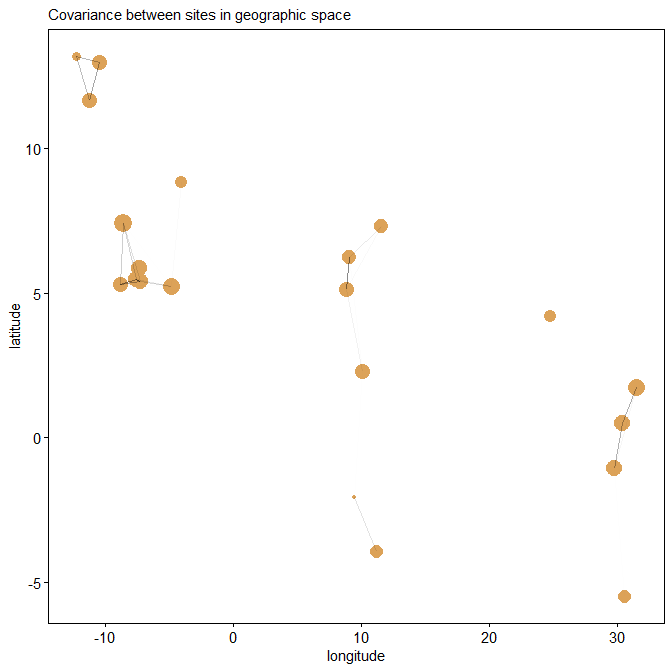


a)

b)

**Figure 8**: Spatial covariance among the 22 sites is evident for sites that a) that the covariance declines to zero after a spatial radius of 300-500 km and b) cluster within a close geographic range. In a),the black line depicts the posterior mean covariance and the thin lines show 100 draws sampled from the posterior distribution, which reveal some uncertainty about the spatial covariance in our data. In b), the sizes of the circles represent the value for the Human footprint for a given site and thicknesses of the lines correspond to the covariance between the connected sites.
